# Conference Reports: FOURTH INTERN A TIONAL SYMPOSIUM ON RESONANCE I ON IZATI ON SPECTROSCOPY AND ITS APPLICA TIONS National Bureau of Standards, Gaithersburg, MD, April 10–15, 1988

**DOI:** 10.6028/jres.094.016

**Published:** 1989

**Authors:** J.P. Young

**Affiliations:** Analytical Chemistry Division, Oak Ridge National Laboratory, Oak Ridge, TN 37831

The technique of resonance ionization spectroscopy (RIS), makes use of the resonant absorption of photons via allowed electronic transitions to transfer an electron in a neutral gaseous atom (or molecule) from some initial state through various excited states to the continuum. This process can be elementally, even isotopically, selective. The product is an ion pair, and either the electron or the cation of the pair can be detected.

The present symposium is the latest in a biannual series of meetings dealing with the photophysics of the RIS process in its many forms, with proposed applications and with descriptions of developed applications. It is quite apparent that, over the course of these four symposia, emphasis of the meetings has shifted from the fundamentals and proposed experiments to actual applications. A considerable effort is also now underway on molecular RIS. This aspect of the field has seen a significant growth from the last meeting as evidenced by the increase in the number of papers on this topic.

A number of interesting and timely papers were presented, but only a few selected highlights can be described here. That RIS techniques are presently in use for acquiring real analytical results was evident in the work presented by R. Willis and coworkers at Atom Sciences, Inc. (Oakridge, TN) dealing with the determination of very low amounts of ^81^Kr in ground water samples. These analyses are used to determine the age of such water samples. Modern water contains 1000 atoms of ^81^Kr per liter. From 50-liter samples of water obtained from a location in Canada, about 6000 atoms of ^81^Kr were found. From a comparison of this number to the ^81^Kr found in an equivalent modern air samples, the age of the ground water can be deduced. The sensitivity of this RIS determination of ^81^Kr is well beyond the sensitivity of the established radioactive counting techniques.

In an experiment designed to measvure the fundamental structure of simple atoms and molecules, S. R. Lundeen of the University of Notre Dame detected weakly populated Rydberg states of He and H_2_ by RIS techniques. RIS is the method of choice and really the only technique that can easily produce the information required in this study.

Several papers addressed the ability of RIS to detect and determine transient and/or short-lived species. A particular example of this application involved the study by U. Kronert and coworkers (from the University of Mainz, West Germany) of short-lived ^185^Au and ^184^Au isotopes (half-lives of 4.3 min and 53 s, respectively), generated and implanted in graphite. About 60% of some 5×10^9^ atoms were laser desorbed from the foil and then resonantly ionized and mass analyzed by a three-color RIS process. As one result of this experiment, hyperfine splitting and isotope shift information was obtained for the isotope investigated.

A paper by G. Bekov, from the U.S.S.R. Academy of Sciences, was of particular interest. He presented a description of their laser process that involves optical excitation followed by electric field ionization. Their studies have been applied to ultrasensitive detection of elements of geochemical interest. Their results support the theory of Alvarez for an extraterrestrial origin of the Cretaceous-Tertiary boundary.

The RIS technique has now been sufficiently studied so that realistic assessment of the current limitations can be made. Papers and posters dealing with this aspect were presented. Two points might be emphasized here. The sample matrix does influence the magnitude and composition of the evolved species that are required for RIS study. This point was brought out in the third symposium and became more apparent in this meeting. Matrix effects do not destroy the usefulness of the technique, but they do need to be addressed in analytical applications. A second problem, that is becoming more obvious as the precision of the technique improves, is that of bias in isotope ratio measurements carried out by resonance ionization mass spectrometry (RIMS). These bias effects appeared in results of several studies and were discussed formally and informally during the meeting. It seems obvious that further evaluation of these effects will be made, and the results will appear in the literature and be presented in the fifth symposium scheduled to be held in Ispra, Italy, in 1990.

The symposium was truly international. Over 150 participants attended from nearly 20 countries, including the U.S.S.R. for the first time. It was funded by a number of organizations: Office of Health and Environmental Research of the U.S. Department of Energy, U.S. Air Force Office of Scientific Research, Battelle Pacific-Northwest Laboratories, EG&G Corporation, Department of Commerce, National Bureau of Standards, National Science Foundation, and the University of Tennessee. The detailed proceedings has been published by the Institute of Physics, Techno House, Bristol, U.K., under the title "Resonance Ionization Spectroscopy 1988,” Conference Series No. 94.

